# 

**Published:** 1916-07

**Authors:** 


JULY, 1916. SPECIAL NUMBER.

Vol. XXXIV. No. 130.

### THE BRISTOL MEDICO-CHIRURGICAL JOURNAL

*PUBLISHED UNDER THE AUSPICES OF*

*THE BRISTOL MEDICO-CHIRURGICAL SOCIETY.*

*Editor:* P. WATSON-WILLIAMS, M.D.

WITH WHOM ARE ASSOCIATED

J. MICHELL CLARKE, M.A., M.D., LL.D.

J. LACY FIRTH, M.S., M.D., JAMES SWAIN, M.S., M.D.

J. A. NIXON, B.A., M.D., *Assistant Editor.*

*Acting Editorial Secretary:* W. A. SMITH, M.A., M.B.

BRISTOL: J. W. ARROWSMITH LTD.

London: Simpkin, Marshall, Hamilton, Kent & Co. Ltd.

*Price Three Shillings net.*

